# Electromyographic activity of equine abdominal muscles during single and double riding in hippotherapy

**DOI:** 10.7717/peerj.21317

**Published:** 2026-05-22

**Authors:** Liz R. S. de Lima, Cesar F. Amorim, Marcus Fraga Vieira, Emmanuel Arnhold, Robson D. Scoz, Luciana R. G. Brandstetter, Lara C. S. Costa, Kate M. C. Barcelos

**Affiliations:** 1 Veterinary and Zootechnics School, Universidade Federal de Goiás, Goiânia, Goiás, Brazil; 2 Laboratory of Physical and Functional Analysis in Physiotherapy (LAFFFi), Egas Moniz Interdisciplinary Research Center (CiiEM), Egas Moniz School of Health and Science, Almada, Setubal, Portugal; 3 Physical Therapy Department, Human Performance Laboratory, Florida International University, Miami, Florida, United States; 4 Lab Corinthians R9, Sport Club Corinthians Paulista, São Paulo, São Paulo, Brazil; 5 BioNR Research Laboratory, Quebec University, Saguenay, Canada; 6 Master and Doctoral Programs in Physical Therapy, Universidade de São Paulo, São Paulo, São Paulo, Brazil; 7 Bioengineering and Biomechanics Laboratory, Universidade Federal de Goiás, Goiânia, Goiás, Brazil

**Keywords:** Hippotherapy, Equine therapy, Surface electromyography, Horse biomechanics, Abdominal muscles, Rider load, Equine welfare

## Abstract

**Background:**

Hippotherapy is a therapeutic intervention that uses the horse as a biomechanical and sensorimotor agent to promote rehabilitation in individuals with physical and neurological disabilities. Depending on patient needs, riding may be performed with a single rider or with double riding, in which a therapist accompanies the practitioner on the horse. However, the effects of these riding configurations on equine trunk muscle activity remain poorly understood, despite their relevance for both horse welfare and the quality of therapeutic stimuli delivered to the patient.

**Methods:**

Nine clinically healthy adult horses (mean body mass: 388 kg) routinely employed in hippotherapy were evaluated. Two healthy female physiotherapists trained in hippotherapy (26 and 27 years; 50.9 and 51.3 kg) served as riders in all experimental conditions. Surface electromyography (sEMG) was recorded bilaterally from the Rectus Abdominis (RA) and External Oblique Abdominis (EOA) muscles during walking at a constant speed of 1.43 m/s under three randomized conditions: without rider (WR), single rider (R1), and double riding (R2). Each trial consisted of a 10-s stable walking period. Data were analyzed using randomized block ANOVA with Tukey’s *post hoc* tests and effect size estimates (Cohen’s *d* and Hedges’ *g*).

**Results:**

Compared with WR, double riding (R2) resulted in significantly greater activation of both RA and EOA muscles bilaterally (*p* < 0.05), with large effect sizes (*g* = 1.50–2.52). No significant differences were observed between R1 and WR when rider load did not exceed 14.75% of the horse’s body weight. Comparisons between R2 and R1 revealed asymmetric activation patterns, particularly increased activation of the left EOA and right RA muscles (*p* < 0.05; large effect sizes), suggesting compensatory neuromuscular strategies to maintain trunk stability under increased load and movement.

**Conclusions:**

Single riding with light loads does not substantially alter equine abdominal muscle activity during walking. In contrast, double riding increases muscular demand and induces asymmetric activation patterns, which may reflect compensatory mechanisms related to load magnitude, rider movement, and handling configuration. These findings have important implications for equine welfare and for optimizing therapeutic decision-making in hippotherapy practice.

## Introduction

Hippotherapy is a therapeutic intervention that uses the horse as a dynamic and biomechanical agent to promote functional, sensory, and neuromotor rehabilitation in individuals with physical, neurological, and developmental disabilities (Brasil; Majewski & de Oliveira, 2020; Caobianco et al., 2019). The rhythmic and repetitive tridimensional movement of the horse’s gait provides afferent stimuli to the practitioner that, in some extension, resemble that provided by human walking, facilitating postural control, balance, muscle activation, and sensorimotor integration in the practitioner (Cherng et al., 2004; Hildebrand, 1965; Clayton, 1995). As a result, hippotherapy has been widely applied in populations such as children with cerebral palsy, individuals with autism spectrum disorder, and patients with neurological impairments (Luz, Boaretto & Rodrigues, 2018; Valentin & Zsoldos, 2016; Wijnberg & Franssen, 2016).

According to Brazilian Law No. 13.830/2019 and the operational framework adopted by the National Equine Therapy Association of Brazil (ANDE-Brasil), equine therapy encompasses different intervention programs that vary according to the functional capacity of the practitioner (Suzuki, 2020). Within this framework, hippotherapy represents a clinical modality focused on health care, in which the horse is used as a kinesiotherapeutic resource for individuals who are unable to maintain independent postural control while riding. As the practitioner’s functional abilities improve, equine therapy may progress to educational and re-educational programs, and eventually to basic horseback riding, characterized by greater autonomy and voluntary control of the horse. In the present study, these modalities are treated as distinct stages rather than synonymous terms, and the focus is restricted to hippotherapy riding conditions.

During hippotherapy, riding may be performed with a single rider or with double riding, in which a therapist accompanies the practitioner on the horse to provide postural assistance and ensure safety (Back & Clayton, 2013). Although double riding may be clinically indicated for individuals with severe motor impairments, it is contraindicated in some equine therapy centers due to concerns regarding increased mechanical and physiological demands imposed on the horse. Previous biomechanical and veterinary studies have demonstrated that increases in rider load alter the horse–rider system’s center of mass, increase vertical forces acting on the spine, and may negatively affect gait symmetry and locomotor regularity (Zadnikar & Kastrin, 2011; Lucena-Antón, Rosety-Rodríguez & Moral-Munoz, 2018; Barsanti, 2019; McGowan, Stubbs & Jull, 2007). Moreover, equine welfare guidelines commonly recommend that the total mounted load should not exceed approximately 17–20% of the horse’s body weight, as higher load ratios are associated with greater risk of fatigue, discomfort, and musculoskeletal strain (Haussler, 1999; Zsoldos et al., 2010a; Scheven, 2010). In this context, double riding represents a relevant scenario in which both total load and load distribution may influence the horse’s neuromuscular response.

Electromyography (EMG) is a well-established method for assessing muscle activation patterns during dynamic tasks and has been increasingly applied in equine biomechanics to investigate locomotor control and load-related adaptations (Zsoldos et al., 2018, 2010b; Powell et al., 2008). In horses, surface electromyography (sEMG) offers a non-invasive approach for evaluating superficial muscles during movement, making it suitable for studies involving therapeutic and clinical settings. Changes in load magnitude, rider movement, and handling configuration may alter the amplitude and symmetry of electromyographic signals, reflecting neuromuscular strategies adopted by the horse to maintain trunk stability and gait quality under varying mechanical demands (Matsuura et al., 2013; Robert, Valette & Denoix, 2001; De Luca, 1997).

The equine trunk musculature plays a central role in stabilizing the spine and transferring forces between the limbs during locomotion. Among these muscles, the hypaxial abdominal muscles are particularly relevant for trunk stabilization and postural control (Soderberg & Cook, 1984). The Rectus Abdominis (RA) contributes to ventral trunk support and spinal flexion, while the External Oblique Abdominis (EOA) assists in lateral stabilization and rotational control of the trunk during gait (McManus et al., 2021; R Core Team, 2023). These muscles are also anatomically superficial, allowing reliable assessment using sEMG during mounted conditions. In contrast, deeper or epaxial muscles, such as the longissimus dorsi and multifidus, are less accessible to surface electrodes and are directly covered by the rider during hippotherapy, limiting the feasibility and reliability of their assessment using sEMG in this context.

Despite the growing use of hippotherapy worldwide, limited objective data are available regarding how different riding configurations affect equine muscle activity, particularly under conditions that may challenge horse welfare. Most existing studies have focused on kinematics, ground reaction forces, or rider outcomes, with relatively little attention given to the neuromuscular responses of the horse itself (Arnhold, 2013; Gracovetsky & Iacono, 1987; Dvořáková et al., 2009). Understanding how single and double riding influence equine trunk muscle activation may provide valuable insights into compensatory strategies adopted by the horse, potential sources of asymmetry, and implications for both animal welfare and the consistency of therapeutic stimuli delivered to practitioners.

Therefore, the aim of this study was to compare the electromyographic activity of the RA and EOA muscles in horses during walking under three conditions: without rider, single riding, and double riding during hippotherapy. We hypothesized that double riding would result in increased and asymmetric abdominal muscle activation compared with single riding and walking without a rider, reflecting greater neuromuscular demand associated with increased load and rider movement.

## Methods

### Study design

This was an experimental, within-subjects study designed to compare equine abdominal muscle activity under three walking conditions: without rider (WR), single riding (R1), and double riding (R2) during hippotherapy. Each horse served as its own control, and the order of the experimental conditions was randomized to minimize potential order effects.

### Animals

The use of animals in the activities carried out in this experiment was approved by the Ethics Committee for the Use of Animals at the Federal University of Goiás (CEUA-UFG), under protocol number 004/20. The sample size calculation was based on previously published experimental studies on the electromyographic analysis of horses’ trunk muscles during gait (Zsoldos et al., 2010a, 2010b). Nine animals were used: 4 mixed breed (SRD), 2 Crioulos (C), 1 Arabian (A), 1 Thoroughbred (PSI) and 1 Mangalarga Marchador (MM) from the National Equine Therapy Association (ANDE-Brasil), eight males and one female (SRD), with an average age of 8 years (from 5 to 17 years) and an average weight of 388 kgs (345 to 450 kgs), the conformation data of the animals are shown in Table 1.

**Table 1:** Morphometric characteristics of the sample (means ± standard deviation).

Sample (*n* = %)	
Male (*n* = %)	8 = 88.8%
Female (*n* = %)	1 = 11.2%
Total (*n* = %)	9 = 100%
Morphometric data	
Age (years)	8.03 ± 3.11
Height (cm)	145.83 ± 7.82
Body mass (kg)	388.96 ± 81.68
Heart girth (cm)	170.84 ± 4.21
Cannon diameter (cm)	0.19 ± 0.015
Body length (cm)	149.82 ± 4.48
Withers height (cm)	148.21 ± 3.53
Croup height (cm)	146.80 ± 4.11

All the horses included in this study were clinically healthy and were assessed by an experienced veterinarian, showing no asymmetries or lameness. All horses had been working in equine therapy for a minimum of 3 years. During a typical working week, horses perform equine therapy sessions approximately three times per week, with 2 to 4 sessions per day, each session lasting 30 min, alternating periods of walking and stationary tasks. In the week of the experiment, however, the horses did not participate in regular sessions. For physical maintenance, these animals also perform basic riding, a function that is utilized in some phases of the equine therapy program.

### Riders

Two experienced and physically fit riders rode all the animals. Both riders were healthy female physiotherapists trained in hippotherapy (26 and 27 years old; body weights: 50.9 and 51.3 kgs; heights: 1.58 and 1.60 m). They had basic experience with horses and more than 2 years of clinical experience delivering hippotherapy sessions. The same two individuals were used in all trials and conditions. The ratio between horses and riders can be seen in Table 2.

**Table 2:** Individual horse body weight and relative rider load calculations.

ID	HBW (kg)	CALC20	L20 (kg)	CALC29	L29 (kg)	SRL (%)	DRL (%)
Horse 1	345	345 × 20%	69.0	345 × 29%	100.05	14.75	29.82
Horse 2	385	385 × 20%	77.0	385 × 29%	111.65	13.22	26.54
Horse 3	450	450 × 20%	90.0	450 × 29%	130.50	11.31	22.86
Horse 4	370	370 × 20%	74.0	370 × 29%	107.30	13.75	27.62
Horse 5	440	440 × 20%	88.0	440 × 29%	127.60	11.56	23.22
Horse 6	385	385 × 20%	77.0	385 × 29%	111.65	13.22	26.54
Horse 7	367	367 × 20%	73.4	367 × 29%	106.43	13.86	27.85
Horse 8	385	385 × 20%	77.0	385 × 29%	111.65	13.22	26.54
Horse 9	365	365 × 20%	73.0	365 × 29%	105.85	13.94	28.00
Mean	388	—	77.6	—	112.52	13.11	26.34
Maximum	450	—	90.0	—	130.50	11.31	22.71
Minimum	345	—	69.0	—	100.05	14.75	29.62

Note:

The percentage of horse body weight is presented prior to each corresponding absolute load value. Rider load percentages were calculated using a proportional rule in which horse body weight was considered 100% and rider weight corresponded to *X*. A single rider body weight of 50.9 kg and a combined double-rider body weight of 102.2 kg were used for all calculations. Abbreviations: HBW, Horse Body Weight; CALC20, calculation used to determine 20% of horse body weight; L20, load corresponding to 20% of horse body weight; CALC29, calculation used to determine 29% of horse body weight; L29, load corresponding to 29% of horse body weight; SRL, Single Rider Load expressed as percentage of horse body weight (50.9 kg); DRL, Double Rider Load expressed as percentage of horse body weight (102.2 kg).

### Location and experimental conditions

The experiment was conducted at the National Equine Therapy Association (ANDE-Brazil) in Brasília, Brazil. All standard procedures were carried out in a location widely used for equine therapy sessions, where the animals were already accustomed to their surroundings. All the horses were led by a guide (always the same person, familiar with the animals) who stood to the left of the horse. The handler was a riding instructor with 6 years of experience and had prior training to maintain a consistent walking rhythm during experimental procedures. The animals were then ridden in a straight line, at walk, on a covered track, on sandy ground with non-woven fabric (TNT) in the following randomized configurations: without a rider, with a rider (single riding), and with two riders (double riding).

The animals warmed up by walking on a 30 × 13 m track covered with sand and TNT flooring before collecting data using the electromyograph. Warm-up consisted of 6 min at walk (approximately 3 min in each direction of the arena). Data collection began manually by visually observing the moment the horse passed the pre-defined track and ended after 10 s of walking at a constant speed of 1.43 m/s, always led in a straight line by the same guide and on the left side, during three sequences: without rider (basal-WR), single (1) rider (R1) and double (2) riders (R2), randomized. The selected walking speed (1.43 m/s) reflects a medium walk commonly used in hippotherapy sessions and was chosen to reduce variability in gait and rider motion (Robert, Valette & Denoix, 2001). The baseline values (WR) in this study were the muscle activity values obtained from horses already in motion, walking at a steady pace without load, and in a straight line with no rider or weight on their back. The time of day for data collection ranged from 15:00 to 18:00, and the order of the animals was randomized. Average ambient temperature during testing was approximately 29 °C.

According to Powell et al. (2008), the most common load recommendation would be 20% of the animal’s live weight (BW). Matsuura et al. (2013), evaluating native Japanese horses, observed that horses could carry up to 29% of their body weight at a trot. Although they were unable to determine the load capacity at a walk, they suggested that horses working only at a walk could reach loads of up to 130 kg. As the stride is the primary gait in equine therapy, we evaluated the weight that the animals carried in this experiment, comparing it to previous reports (Powell et al., 2008; Matsuura et al., 2013). The live weight of the animal was compared to the live weight of the riders in R1 and R2 to determine an ideal load ratio and assess the possible influence of the load on electromyographic activity.

### Electromyography

The SAS2000V6-WF signal acquisition system captured the electromyographic data and transducers from EMG System do Brazil LTDA, which consisted of a 6-channel module, a signal conditioner with a band-pass filter with a cut-off frequency of 20 to 500 Hz, and a common standard mode rejection rate of >120 dB. The signals were acquired using a 16-bit A/D converter with an anti-aliasing sampling frequency of 2 kHz for each channel and an input range of ±5 mV. All data was processed using proprietary acquisition and analysis software (EMGLab).

The animal’s skin at each electrode attachment site was trichotomized and sanitized with gauze soaked in 2% chlorhexidine degermant, then cleaned with water and dried with a paper towel to ensure better adherence of the surface electrodes. The electrodes were positioned bilaterally parallel to the direction of the muscle fibers of each muscle, with an inter-electrode distance of 3 cm (Robert, Valette & Denoix, 2001), and were reinforced with hypoallergenic adhesive tape. The RA and EOA muscles were analyzed. They were located and identified by non-invasive palpation of anatomical landmarks, and their location was confirmed by ultrasound. Active bipolar surface electrodes were used to reduce the effects of electromagnetic interference and other noise, consisting of two Ag/AgCl surface electrodes. The point of attachment of the electrodes on the RA was 6 cm from the umbilical scar (Robert, Valette & Denoix, 2001) and on the EOA at the level of the 16th rib (Zsoldos et al., 2010a). Electrode placement was visually monitored during walking, and analysis prioritized segments with stable signals and minimal visible motion artifacts.

The sEMG signal was mathematically rectified using a Butterworth linear wrapper filter (4th order), at a rate of 5 Hz, normalized on a time and amplitude basis (De Luca, 1997; Soderberg & Cook, 1984). From this, the average (RMS) of the signal was taken. In all instrumented procedures, the sEMG signals were captured and analyzed according to the recommendations of the International Society of Electrophysiology Kinesiology (ISEK) (McManus et al., 2021). The evaluation recorded 10 s of electromyographic signal from the RA and EOA muscles bilaterally. The 10-s window corresponded to steady-state walking (excluding gait initiation), comprising approximately eight stride cycles; when necessary, segments were selected manually to ensure a complete 10-s interval without visible interference or signal instability.

### Statistical analysis

To assess significant differences between the means, an analysis of variance (ANOVA) was conducted using a randomized block design (with each animal in a block) and followed by Tukey’s test. The Shapiro-Wilk test was used to assess the normality of the data. The homogeneity of variance was evaluated using Bartlett’s test. The significance level (*p*) considered was 0.05. The R software (R Core Team, 2023) and the R easy ANOVA package (Arnhold, 2013) were used to carry out the analyses. The results were evaluated by comparing the muscles within each block (intra-group) and between the treatments (inter-group) in this study. In addition, effect sizes were calculated for the pairwise contrasts using Cohen’s d and Hedges’ g. The values taken as reference were 0.1, 0.4, and 0.8 for small, medium, and large effect sizes, respectively (Zieliński, 2025).

## Results

All horses completed the experimental protocol without adverse events. Electromyographic signals with adequate quality were obtained for all muscles and conditions analyzed.

### External oblique abdominis (EOA)

For the left EOA muscle, significant differences were observed between experimental conditions. Double riding (R2) resulted in significantly greater electromyographic activity compared with single riding (R1) (*p* = 0.0367), with a large effect size (Cohen’s d = 1.31; Hedges’ g = 1.24). When compared with walking without rider (WR), R2 also demonstrated significantly higher activation (*p* = 0.0024), with a large effect size (d = 1.98; g = 1.88). No significant difference was observed between R1 and WR (*p* = 0.3576), despite a medium effect size (d = 0.66; g = 0.63).

For the right EOA, R2 showed significantly greater activation compared with WR (*p* = 0.0123), with a large effect size (d = 1.58; g = 1.50). Comparisons between R2 and R1 (*p* = 0.2646) and between R1 and WR (*p* = 0.2321) did not reach statistical significance, although medium effect sizes were observed for both contrasts (R2 *vs*. R1: d = 0.77; g = 0.73; R1 *vs*. WR: d = 0.81; g = 0.77).

### Rectus abdominis (RA)

For the left RA muscle, double riding (R2) resulted in significantly greater activation compared with WR (*p* = 0.0042), with a large effect size (d = 1.84; g = 1.75). The comparison between R2 and R1 approached statistical significance (*p* = 0.0633) and demonstrated a large effect size (d = 1.17; g = 1.11). No significant difference was observed between R1 and WR (*p* = 0.3588), with a medium effect size (d = 0.66; g = 0.63).

For the right RA, a significant differences were identified across conditions. R2 demonstrated significantly greater activation compared with R1 (*p* = 0.0059), with a large effect size (d = 1.75; g = 1.66). Comparisons between R2 and WR also revealed significantly higher activation in R2 (*p* = 0.0002), with a large effect size (d = 2.65; g = 2.52). No significant difference was found between R1 and WR (*p* = 0.1751), despite a large effect size (d = 0.89; g = 0.85) (Table 3).

**Table 3:** Means contrasts, Tukey probability values e effect size using Cohen d and Hedges g mehods.

Variable	Contrast	p (Tukey)	d (Cohen)	g (Hedges)
Left EOA	R2-R1 = 1.6111	0.0367	1.31	1.24
	R2-WR = 2.4333	0.0024	1.98	1.88
	R1-WR = 0.8222	0.3576	0.66	0.63
Right EOA	R2-R1 = 1.6666	0.2646	0.77	0.73
	R2-WR = 3.4222	0.0123	1.58	1.5
	R1-WR = 1.7556	0.2321	0.81	0.77
Left RA	R2-R1 = 1.6777	0.0633	1.17	1.11
	R2-WR = 2.6333	0.0042	1.84	1.75
	R1-WR = 0.9556	0.3588	0.66	0.63
Right RA	R2-R1 = 2.4444	0.0059	1.75	1.66
	R2-WR = 3.6889	0.0002	2.65	2.52
	R1-WR = 1.2445	0.1751	0.89	0.85
WR.RA	RIGHT-LEFT = 1.1555	0.0085	1.63	1.55
R1.RA	RIGHT-LEFT = 1.3333	0.0092	1.6	1.52
R2.RA	RIGHT-LEFT = 2.1	0.0319	1.22	1.16

### Side-to-side comparisons

Side-to-side comparisons revealed significant asymmetries in Rectus Abdominis activity across conditions. During walking without rider (WR), the right RA demonstrated significantly greater activation than the left RA (*p* = 0.0085), with a large effect size (d = 1.63; g = 1.55). Similar asymmetry was observed during single riding (R1), with higher activation on the right side (*p* = 0.0092; d = 1.60; g = 1.52). During double riding (R2), the magnitude of this asymmetry increased, with the right RA exhibiting significantly greater activation than the left RA (*p* = 0.0319), accompanied by a large effect size (d = 1.22; g = 1.16).

### Summary of main findings

Overall, double riding (R2) consistently resulted in higher electromyographic activity of equine abdominal muscles compared with walking without rider and single riding, with predominantly large effect sizes. In contrast, single riding (R1), when the total load did not exceed 14.75% of the horse’s body weight, did not produce statistically significant differences in abdominal muscle activity compared with walking without rider.

## Discussion

The present study investigated the electromyographic activity of equine abdominal muscles during walking under three hippotherapy-related conditions: without rider, single riding, and double riding. The main findings indicate that double riding significantly increases abdominal muscle activation compared with both walking without rider and single riding, with predominantly large effect sizes. In contrast, single riding, when total load remained below 14.75% of the horse’s body weight, did not produce statistically significant changes in abdominal muscle activity compared with walking without rider. These findings partially confirm our initial hypothesis and provide objective evidence regarding the neuromuscular demands imposed on horses during different hippotherapy riding configurations.

The increase in RA and EOA activation observed during double riding is consistent with previous biomechanical evidence demonstrating that increases in rider load alter the horse–rider system’s center of mass and increase mechanical demands on the trunk musculature (Zadnikar & Kastrin, 2011; Lucena-Antón, Rosety-Rodríguez & Moral-Munoz, 2018; Barsanti, 2019; McGowan, Stubbs & Jull, 2007). Abdominal muscles play a central role in stabilizing the equine trunk during locomotion by counteracting spinal extension moments and assisting in lateral and rotational control (Soderberg & Cook, 1984; McManus et al., 2021; R Core Team, 2023). The large effect sizes observed in the present study suggest that the neuromuscular response to double riding is not only statistically detectable but also biomechanically meaningful, reflecting a substantial increase in muscular demand.

Interestingly, single riding did not result in significant changes in abdominal muscle activity when compared with walking without rider, despite medium effect sizes in some contrasts. This finding aligns with previous recommendations indicating that loads below approximately 15–20% of the horse’s body weight are generally well tolerated at a walk and may not substantially disrupt locomotor patterns (Powell et al., 2008; Matsuura et al., 2013). From a practical standpoint, these results support the notion that light single-rider loads, as commonly used in hippotherapy, do not impose excessive neuromuscular demands on the horse’s abdominal musculature.

A notable finding of this study was the presence of asymmetric activation patterns, particularly during double riding. Increased activation of the left EOA and right RA muscles suggests that horses adopt compensatory neuromuscular strategies to maintain trunk stability under conditions of increased and asymmetrically distributed load. Such asymmetries may be influenced by multiple factors, including rider positioning, subtle differences in rider movement, and the presence of a handler consistently positioned on the left side of the horse. Previous studies have reported that asymmetric loading and handling can influence equine muscle activation and gait regularity (Barsanti, 2019; McGowan, Stubbs & Jull, 2007), supporting the interpretation that double riding introduces additional stabilization challenges beyond those associated with load magnitude alone.

The choice to evaluate the RA and EOA muscles was motivated by their functional relevance and feasibility for surface electromyography during mounted conditions. These muscles contribute directly to ventral trunk stabilization and lateral control during gait (Soderberg & Cook, 1984; McManus et al., 2021; R Core Team, 2023), making them particularly sensitive to changes in load and balance. While deeper epaxial muscles such as the longissimus dorsi and multifidus are also critically involved in trunk stability, their anatomical depth and dorsal location limit reliable assessment using surface electrodes, especially during hippotherapy where the rider occupies the dorsal trunk (Zsoldos et al., 2018; Zsoldos et al., 2010b). Therefore, although the present findings do not provide a complete picture of trunk muscle coordination, they offer meaningful insights into the hypaxial muscular response to different riding configurations.

From a clinical perspective, alterations in equine muscle activation patterns may have implications beyond horse welfare alone. Hippotherapy relies on the transmission of rhythmic, symmetrical, and predictable movement stimuli from the horse to the practitioner (Cherng et al., 2004; Hildebrand, 1965; Clayton, 1995). Increased muscular demand and asymmetrical activation patterns in the horse may subtly alter stride quality and trunk motion, potentially influencing the consistency and symmetry of the sensory input delivered to the patient. Although patient outcomes were not assessed in this study, the present findings highlight the importance of considering equine neuromuscular responses when selecting riding configurations, particularly for vulnerable horses or during prolonged therapeutic sessions.

Despite the strengths of this study, several limitations should be acknowledged. First, sample size calculations were based on previous equine EMG studies rather than on expected differences between riding conditions and should therefore be considered indicative rather than definitive. Second, all horses were led from the left side by the same handler, which may have contributed to the observed asymmetries. Third, only superficial abdominal muscles were assessed, and kinematic data were not collected, limiting the ability to directly associate electromyographic findings with specific gait alterations. Fourth, only one walking speed was evaluated, which, although representative of hippotherapy practice, may not capture the full range of locomotor adaptations. Finally, all riders were healthy and experienced female physiotherapists, which limits the generalizability of the findings to situations involving male riders, overweight people, and, particularly, patients with neuromotor disabilities. Furthermore, our results may also be affected by increased exercise exposure time.

Future studies integrating kinematic analyses, deeper trunk musculature assessment, and patient-related outcomes are warranted to further elucidate the interaction between horse biomechanics, therapeutic effectiveness, and long-term equine health.

## Conclusions

This study demonstrates that riding configuration has a meaningful impact on equine abdominal muscle activity during hippotherapy-related walking. These findings indicate that double riding imposes substantially greater neuromuscular demands on the horse, likely reflecting compensatory strategies to maintain trunk stability under increased and asymmetrically distributed load.

Double riding significantly increased abdominal muscle activation and was associated with asymmetric neuromuscular patterns, with predominantly large effect sizes. In contrast, single riding with light loads (≤14.75% of the horse’s body weight) did not produce statistically significant changes in Rectus Abdominis or External Oblique Abdominis activation when compared with walking without a rider.

The results underscore the importance of considering equine neuromuscular responses and load management when selecting riding configurations in hippotherapy with regard to horse welfare. Further studies evaluating the entire movement of horses and patients with neuromotor disabilities under these riding conditions may demonstrate the consistency of therapeutic stimuli, complementing these results.

## Supplemental Information

10.7717/peerj.21317/supp-1 Supplemental Information 1 Supporting Information.

10.7717/peerj.21317/supp-2 Supplemental Information 2 Comparison of the electrical activity means, in RMS, at walk, in the medial portion of the External Oblique Abdominal muscle, between left and right sides without rider, WR, in sigle riding, R1, and double riding, R2. Coefficient of variation; means in microV; means with different letters differ. Graphs from left to right: WR, R1 and R2.

10.7717/peerj.21317/supp-3 Supplemental Information 3 Comparison of the electrical activity means, in RMS, at walk, in the medial portion of the Rectus Abdominal muscle, between left and right sides without rider, WR, in sigle riding, R1, and double riding, R2. Coefficient of variation; means in microV; means with different letters differ. Graphs from left to right: WR, R1 and R2.

10.7717/peerj.21317/supp-4 Supplemental Information 4 Comparison of the electrical activity means, in RMS, at walk, between Double, R2, Single, R1, and Without Rider, WR, in the medial portion of the External Oblique Abdominal muscle on the left and right sides. CV, coefficient of variation; mean values in microV; R2, double riding, R1, single riding, WR, without rider. Averages with the same letter do no differ. Left graph refers to the muscle on the left side and right graph refers to the muscle on the right side.

10.7717/peerj.21317/supp-5 Supplemental Information 5 Comparison of the electrical activity means, in RMS, at walk, between Double, R2, Single, R1, and Without Rider, WR, in the medial portion of the Rectus Abdominal muscle on the left and right sides. CV, coefficient of variation; mean values in microV; R2, double riding, R1, single riding, WR, without rider. Averages with the same letter do no differ. Left graph refers to the muscle on the left side and right graph refers to the muscle on the right side.

10.7717/peerj.21317/supp-6 Supplemental Information 6 ARRIVE checklist.

10.7717/peerj.21317/supp-7 Supplemental Information 7 Raw Data.

## Additional Information and Declarations

### Data Availability

The following information was supplied regarding data availability:

The data is available in the Supplemental File.
