# Supplementary material for: Electromyographic activity of equine abdominal muscles during single and double riding in hippotherapy
Source: PeerJ. 2026 May 22;14:e21317. doi: 10.7717/peerj.21317 (PMC13200663; doi:10.7717/peerj.21317)
Supplement: Supplemental Information 1 [file peerj-14-21317-s001.docx]

**Supporting information**

**S1 Table. Morphometric characteristics of the sample (means ± standard deviation)*.*** Height (at withers) - was taken by placing the ends of the hypometer on the ground, perpendicular to the horizontal plane, and the arm of the metal rod horizontally over the highest point of the withers; Heart Girth - circumference taken from the end of the withers to the withers (sternum); Body length - distance from the tip of the shoulder blade to the tip of the buttock, taken by fixing the arm of the metal rod at the scapulohumeral joint and running the other end of the hypometer until it touches the tip of the buttock (external to the ischium); Canon Diameter- measured at the narrowest point below the knee; Withers Height - vertical distance between the end of the withers and the ground; Croup Height - was obtained by placing the hypometer on the ground, perpendicular to the horizontal plane, and the arm of the metal rod over the central and highest part of the sacral region.

**S2 Table. Each animal weight (kg) compared with each rider’s weight (kg).**

The percentage of horse body weight is presented prior to each corresponding absolute load value. Rider load percentages were calculated using a proportional rule in which horse body weight was considered 100% and rider weight corresponded to *X*. A single rider body weight of **50.9 kg** and a combined double-rider body weight of **102.2 kg** were used for all calculations.

**S3 Table. Means contrasts, Tukey probability values e effect size using Cohen d and Hedges g mehods.**

WR, without rider; R1, single riding; R2, double riding.

**S1 Fig. Comparison of the electrical activity means, in RMS, at walk, in the medial portion of the External Oblique Abdominal muscle, between the left and right sides without rider, in single riding, and double riding.** CV= Coefficient of Variation; mean values in μv; means with different letters differ. Graphs from left to right: WR, R1 and R2.

**S2 Fig. Comparison of the electrical activity means, in RMS, at walk, in the medial portion of the rectus abdominal muscle, between the left and right sides without rider, single riding and double riding.** CV= Coefficient of Variation; mean values in μv; means with different letters differ. Graphs from left to right: WR, R1 and R2.

**S3 Fig. Comparison of the electrical activity means, in RMS, at walk, between the Double, Single and Without Rider, in the medial portion of the External Oblique Abdominal muscle on the Left and Right sides.** CV = Coefficient of Variation; mean values in μv; R2= Double riding; R1= Single riding; WR= Without Rider. Averages with the same letter do not differ. Left graph refers to the muscle on the left side and right graph refers to the muscle on the right side.

**S4 Fig. Comparison of the electrical activity means, in RMS, at walk, between the Double, Single and Without Rider, in the medial portion of the Rectus Abdominal muscle on the Left and Right sides.** CV= Coefficient of Variation; mean values in μv; R2= Double riding; R1= Single riding; WR= Without Rider. Averages with the same letter do not differ. Left graph refers to the muscle on the left side and right graph refers to the muscle on the right side.
