# Supplementary figures and images for: Electromyographic activity of equine abdominal muscles during single and double riding in hippotherapy
Source: PeerJ. 2026 May 22;14:e21317. doi: 10.7717/peerj.21317 (PMC13200663; doi:10.7717/peerj.21317)

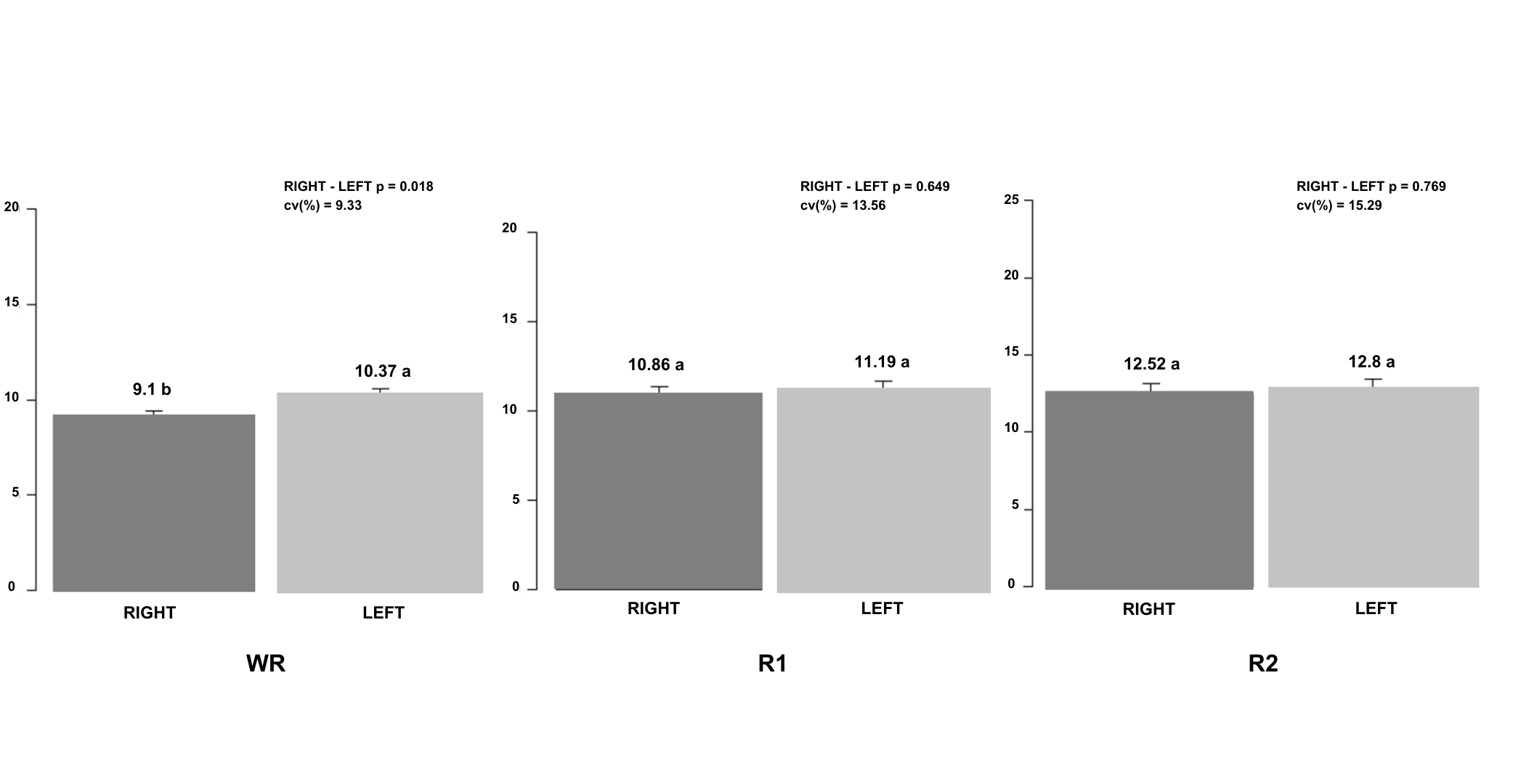

Supplement: Supplemental Information 2 — Coefficient of variation; means in microV; means with different letters differ. Graphs from left to right: WR, R1 and R2. [file peerj-14-21317-s002.png]

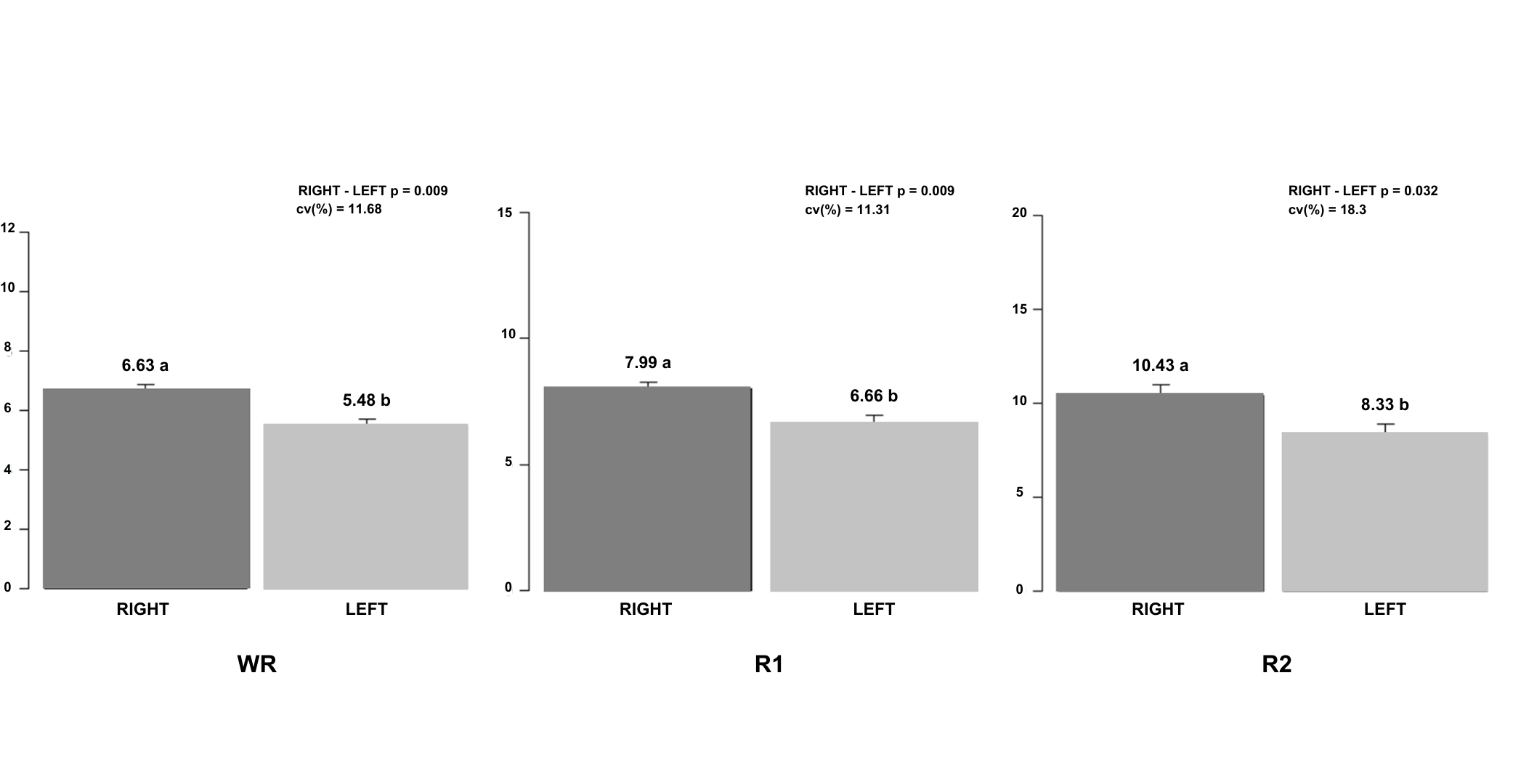

Supplement: Supplemental Information 3 — Coefficient of variation; means in microV; means with different letters differ. Graphs from left to right: WR, R1 and R2. [file peerj-14-21317-s003.png]

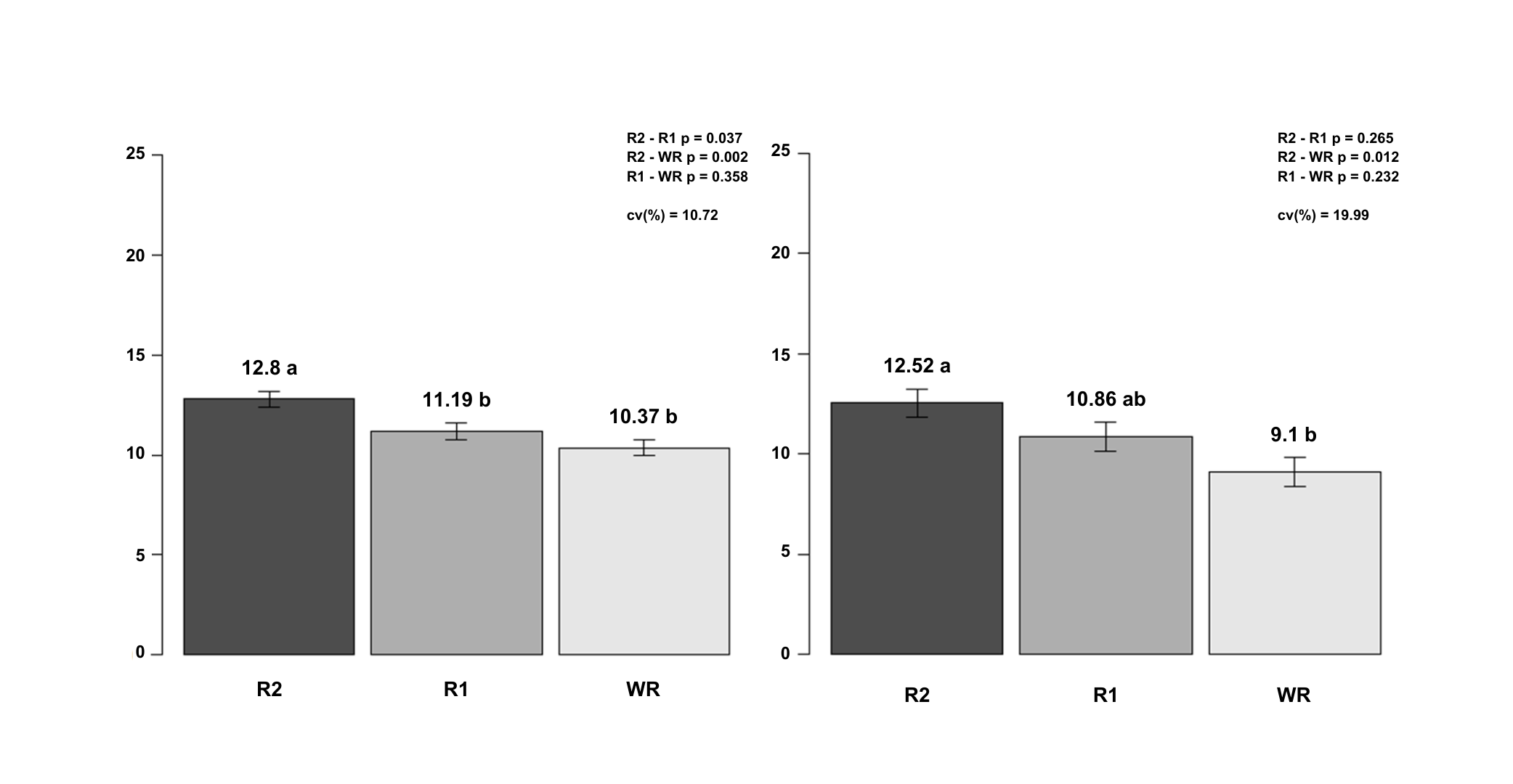

Supplement: Supplemental Information 4 — CV, coefficient of variation; mean values in microV; R2, double riding, R1, single riding, WR, without rider. Averages with the same letter do no differ. Left graph refers to the muscle on the left side and right graph refers to the muscle on the right side. [file peerj-14-21317-s004.png]

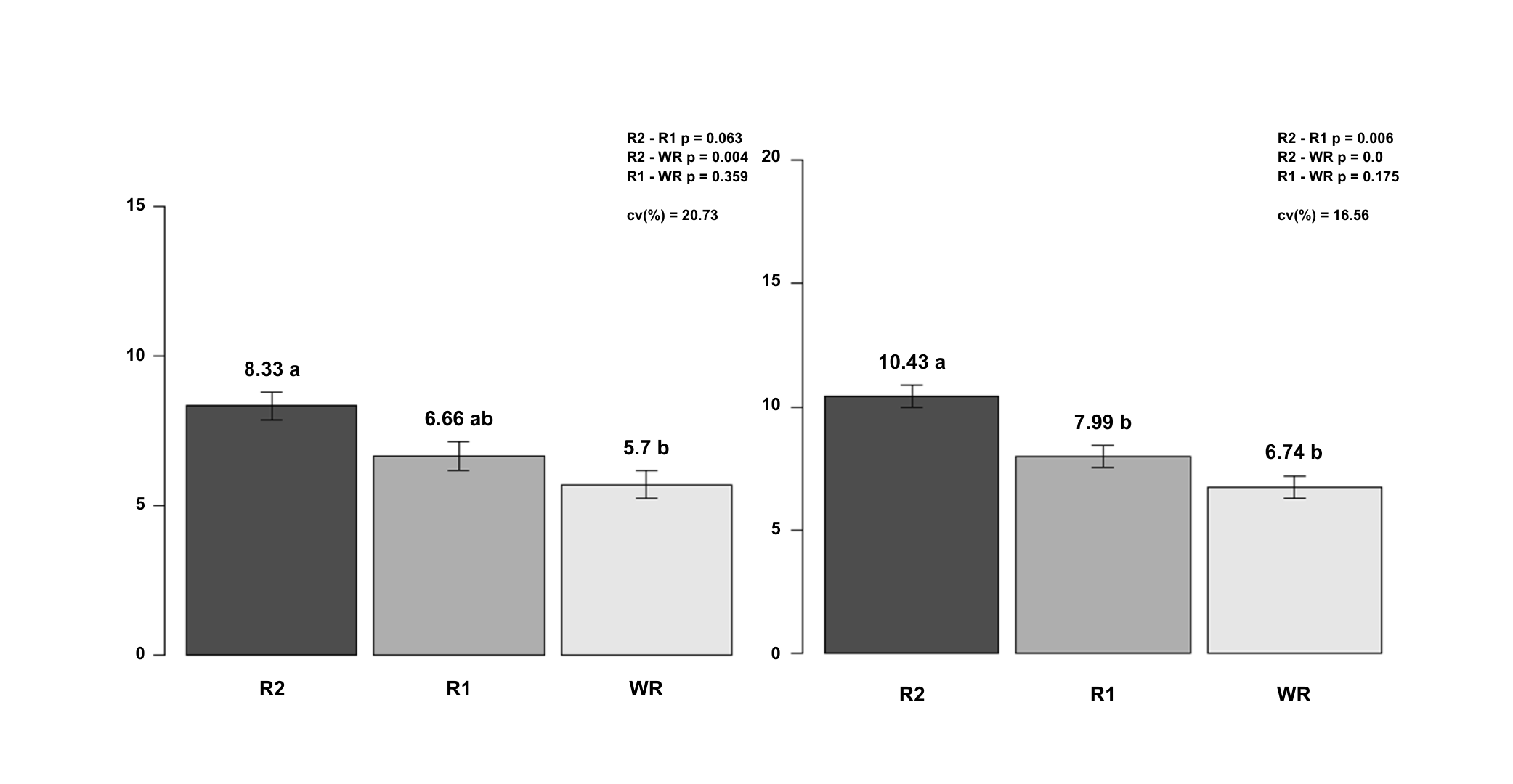

Supplement: Supplemental Information 5 — CV, coefficient of variation; mean values in microV; R2, double riding, R1, single riding, WR, without rider. Averages with the same letter do no differ. Left graph refers to the muscle on the left side and right graph refers to the muscle on the right side. [file peerj-14-21317-s005.png]
